# Numerical Simulation on Self-Propulsion Characteristics of Bionic Flexible Foil Considering Ground Wall Effect

**DOI:** 10.3390/biomimetics9120750

**Published:** 2024-12-10

**Authors:** Yongcheng Li, Nan Zhang, Xinyuan Tang, Ziying Pan, Pengfei Xu

**Affiliations:** 1 Scientific Research Department of Hydrodynamics, China Ship Scientific Research Centre, Wuxi 214082, China; 2 Taihu Laboratory of Deepsea Technological Science, Wuxi 214082, China; 3 College of Oceanography, Hohai University, Nanjing 210024, China

**Keywords:** ground wall effect, self propulsion, travelling motion, fluid–structure interaction

## Abstract

In order to figure out the wall effect on the propulsive property of an auto-propelled foil, the commercial open-source code ANSYS Fluent was employed to numerically evaluate the fluid dynamics of flexible foil under various wall distances. A virtual model of NACA0015 foil undergoing travelling wavy motion was adopted, and the research object included 2D and 3D models. To capture the foil’s moving boundary, the dynamic grid technique coupled with the overlapping grid was utilized to realize the foil’s positive deformation and passive forward motion. The ground wall effect on fluid dynamics (thrust force, lift force and propulsive efficiency) and the flow structures of travelling wavy foil were analyzed. The numerical results show that the existence of the ground wall is beneficial for the propulsive property of foil. Specifically, the existence of the wall can improve the forward speed and efficiency of foil, with a maximum increase of 13% in moving velocity and a 10.5% increase in propulsive efficiency. The conclusions acquired in the current study are of great significance for the design of bionic UUV.

## 1. Introduction

In the natural world, when fishes or birds move close to the wall, some benefits, including drag reduction or lift force enhancement, will be acquired due to the wall effect [1]. For example, little energy will be consumed when hard-boned fish are suspended near the wall [2]. The swimming speed of trout fish can also be improved when the distance between the ground and the trout is small [3]. It is with great regret that the physical mechanism behind the wall effect is still unclear [4,5]. Therefore, there is a pressing need to conduct research on the wall effect to explore its potential applications in biomimetic engineering.

In recent years, the effect of the ground wall on rigid flapping foil has been widely investigated, where the flapping foil undergoes a combined motion of heaving and pitching motion [6,7]. This pioneering work was conducted by Moryossef [8], and his work suggests that the existence of the ground wall leads to an asymmetric phenomenon in lift force. Gao and Lu [9] numerically simulate the process of two-dimensional oval-shaped plates moving towards the ground wall by using the immersed boundary method at *Re* = 200. The simulation results show that three typical stages show up in the process, and they are force enhancement, force reduction, and force recovery, respectively. Furthermore, the overlapping grid technique was adopted by Li [10] to numerically study the effect of the ground wall on the propulsive property of 2D and 3D flapping foil under various Reynolds numbers. The results claim that the effect of the ground wall on 3D foil is less sensitive to 2D foil, and the mutual interaction is closely related to the surrounding vortex structures. Apart from the single flapping foil, taking the ‘bionic bird’ as an example, Su [11] numerically studied the effect of the wall on its fluid dynamics, where the ‘bionic bird’ is composed of the fuselage and two flapping foils. The numerical results show that the lift force of the ‘bionic bird’ is greatly enhanced while the drag force can be reduced under the existence of the ground wall. In addition to this numerical work, some experimental works concerning the wall effect have also been carried out. A foil undergoing pure heaving motion near the solid wall was experimentally studied in a towing tank by Quinn [12]. The experimental results demonstrate that the thrust force can improve by 40%. Troung [13] utilized the Digital Particle Image Velocity (DPIV) to measure the flow structures near the solid wall, and it was found that the reason why the lift force is enhanced lies in the fact that the corresponding leading vortex structures are strengthened due to the existence of the solid wall.

On the other hand, apart from the rigid flapping foil, the flexible oscillating foil [14] is also one of the regular motion manners adopted by natural creatures. In particular, fishes often adopt flexible travelling wavy motion to achieve quick swimming due to the mutual interaction between fishes and their surrounding fluid [15]. Compared to rigid flapping, fewer studies have been conducted on flexible travelling foil considering the ground wall effect. Recently, Li [16] adopted the dynamic grid technique to numerically investigate the ground effect on the hydrodynamic property of flexible foil. The simulation results demonstrated that the existence of a ground wall breaks down the stability along the vertical direction, and the relevant mean value of the lift force exhibits obvious growth. Zhang [17] systematically analyzed the ground effect on the propulsive properties of flexible thread by using direct numerical simulation (DNS). The simulation results show that the closer the ground wall is, the higher the propulsive efficiency of flexible thread will be. Similar work has also been conducted by Dai [18], who also found that the decrease in wall distance is beneficial for the improvement of propulsive properties. The limitation of their work is mainly on low Reynolds number (10^2^), which is much smaller than that of real fish in the natural world. Actually, there exists a huge difference in the ground wall effect when the Reynolds number varies a lot. Xin [19] numerically investigated the flow structures of three-dimensional flexible travelling plates near the wall using the immersed boundary method. The results show that the vortex structures along the chord-wise direction are inhibited, and the energy consumption is also cut down, which may explain why the corresponding propulsive property is improved.

The physical model in the above work concerning the wall effect is stationary; namely, the foil is fixed at a certain position in the flow field, and the incoming velocity is imposed on the inlet of the computation domain [20,21]. However, fishes and birds as creatures all undergo free motion [22,23]. Hence, there must exist a difference between the self-propelled motion and stationary state when investigating the wall effect on oscillating foil. In view of this, the wall effect on the propulsive property of flexible foil undergoing self-propulsion is analyzed of the current study, and the research object includes the 2D model and 3D model. The cross-section of a 3D model can be regarded as 2D, and this 2D model can be seen as a special condition of the 3D model. The conclusions obtained through a two-dimensional model simulation can also provide support for subsequent analysis in the flow mechanism. However, due to the neglect of the wingspan on the two-dimensional model, there is a significant gap between the surface flow and the three-dimensional operating conditions. Specifically, the vortex structure behind the three-dimensional wing is composed of vortex rings on the wing surface and tip vortices on both sides, which is very different from two-dimensional models that rely solely on surface vortex structures. Due to the consideration of the lateral flow mechanism, the fluid dynamic performance of the 3D model is more in line with reality. The overlapping grid technique coupled with the dynamic grid is employed to figure out the wall effect on fluid dynamics and flow structures of flexible oscillating foil under various wall distances. In the rest of this paper, the physical model and motion equations are introduced in Section 2, followed by the numerical method and validation in Section 3. The effect of the ground wall on fluid dynamics and flow structures of 2D and 3D flexible foil are presented and discussed in Section 4. The conclusions acquired in the current study are introduced in Section 5. The novelty of the current study is as follows. For one, the effect of the ground wall on the fluid dynamics of auto-propelled wavy foil is investigated for the first time. In the present literature review, most of the study focuses mainly on fixed travelling wavy foil and little research is conducted on investigating the self-propulsion of travelling wavy foil. For another, the connection between the propulsive property of wavy foil and flow structures is established. A quantitative parameter, the oblique angle, is also put forward to build the connection between the propulsive property of wavy foil and flow structures. In the very near future, a bionic underwater environment based on travelling wavy foils will be designed so it can glide near the seabed. Conclusions acquired in the current study can provide theoretical guidance for the subsequent navigation and control of bionic UUV.

## 2. Physical Model

**Figure 1:**
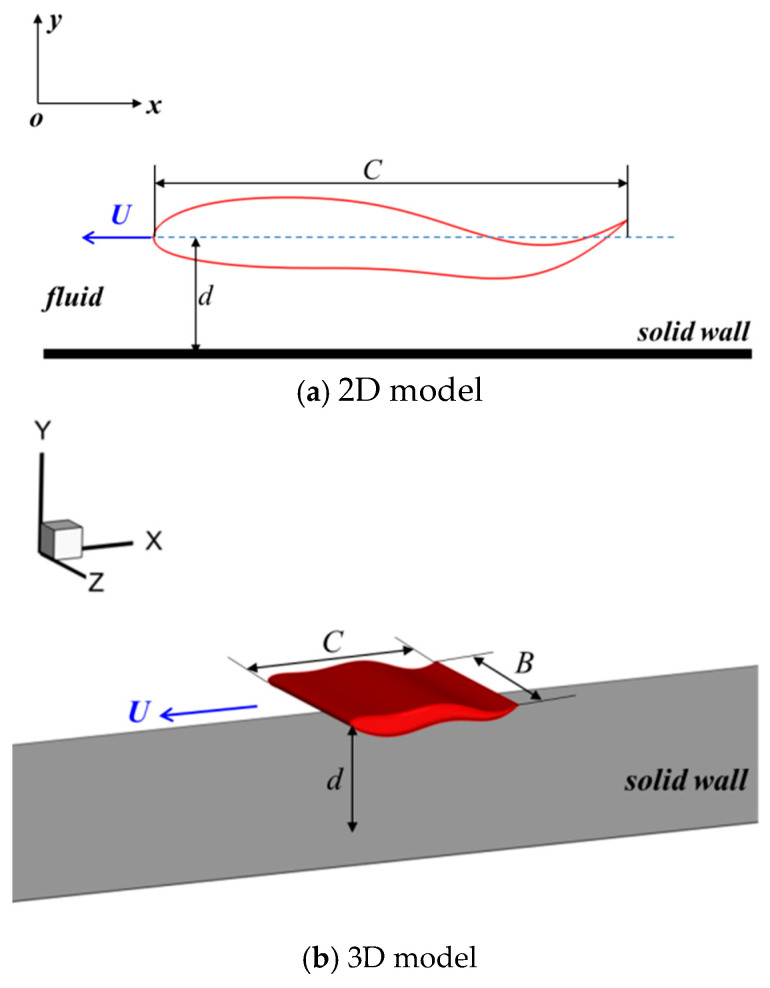
Diagram of physical model.

As shown in Figure 1, the NACA0015 foil is selected as the physical model, and the chord length is set as the characteristic length *C*. The 2D model and 3D model are shown in Figure 1a,b, respectively. The distance between the ground wall and the centre line of the foil is denoted as d; as for the 3D model, the aspect ratio is defined as *AR* = *B*/*C*, where *B* represents the span length. The travelling wavy motion is imposed on the foil, and the relevant expression is written in Formula (1), where *λ* is the travelling wavelength, *f* is the motion frequency, *A*(*x*) is the function of the travelling amplitude, and it is a quadratic function. In the current study, the value of *a_0_* is fixed at 0.02, *a*_2_ = −2*a*_1_.

(1) $$y(x,t)=A(x)\sin(\frac{2\pi }{\lambda }x-2\pi ft)$$

(2) $$A(x)=a_{0}+a_{1}\cdot x+a_{2}\cdot x^{2}$$

**Figure 2:**
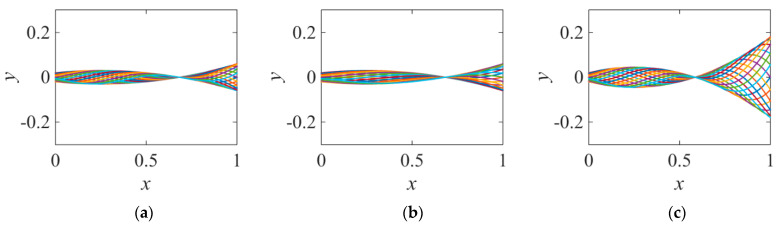
Schematic diagram of foil’s deformation during one complete period under various combinations of motion parameters: (**a**) *λ* = 1.0, *a*_1_ = −0.08, (**b**) *λ* = 2.5, *a*_1_ = −0.08, (**c**) *λ* = 1.0, *a*_1_ = −0.20.

In order to more intuitively demonstrate the process of the travelling wavy motion, the diagram of foil’s deformation during one complete period under various combinations of motion parameters is shown in Figure 2.

## 3. Numerical Method and Validation

### 3.1. Governing Equation

The incompressible Navier–Stokes equation is chosen as the governing equation, and its expression can be written in Formula (3), where **u** and *p* represent the velocity and pressure, respectively. *μ* is the dynamic viscous coefficient, and *ρ* is the fluid density. Considering the fact that the governing equation is not closed, the SST k-ω model is employed to handle the turbulent terms.

(3) $$\nabla \cdot u=0,\;\frac{\partial u}{\partial t}+(u\cdot \nabla )u=-\frac{1}{\rho }\nabla p+\frac{\mu }{\rho }\nabla^{2}u$$

The finite volume approach is adopted to achieve the discretization of Equation (3). The standard wall function is utilized to handle the flow near the wall. The first-order implicit method is utilized to deal with the time term in the governing equation; the second up-wind is adopted to cope with the convection term. The coupling of pressure and velocity is solved using the SIMPLE method.

### 3.2. Computation Domain and Grid

**Figure 3:**
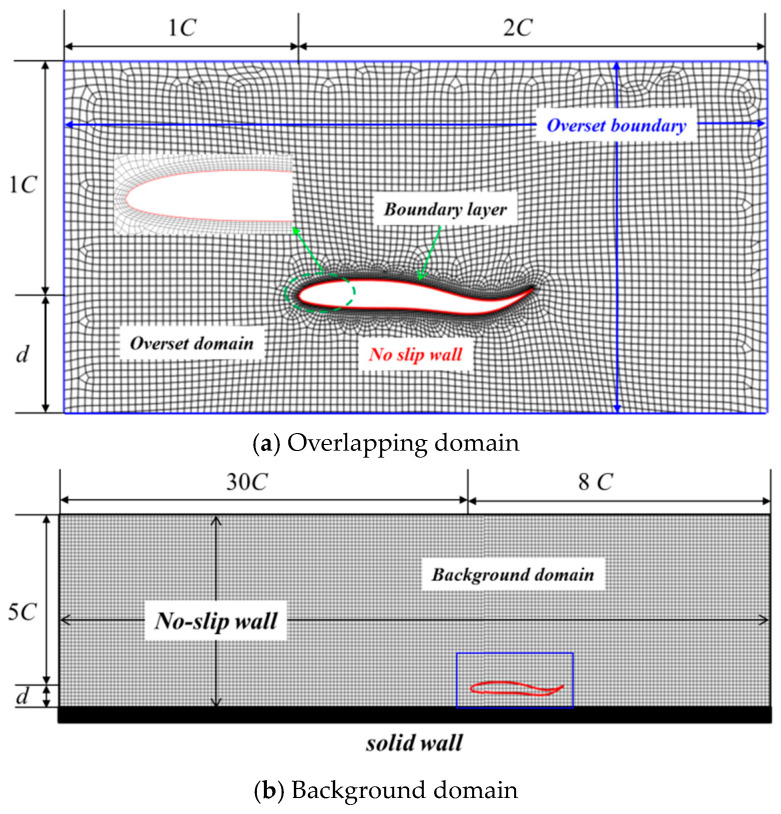
Diagram of computation domain and grid for 2D model.

**Figure 4:**
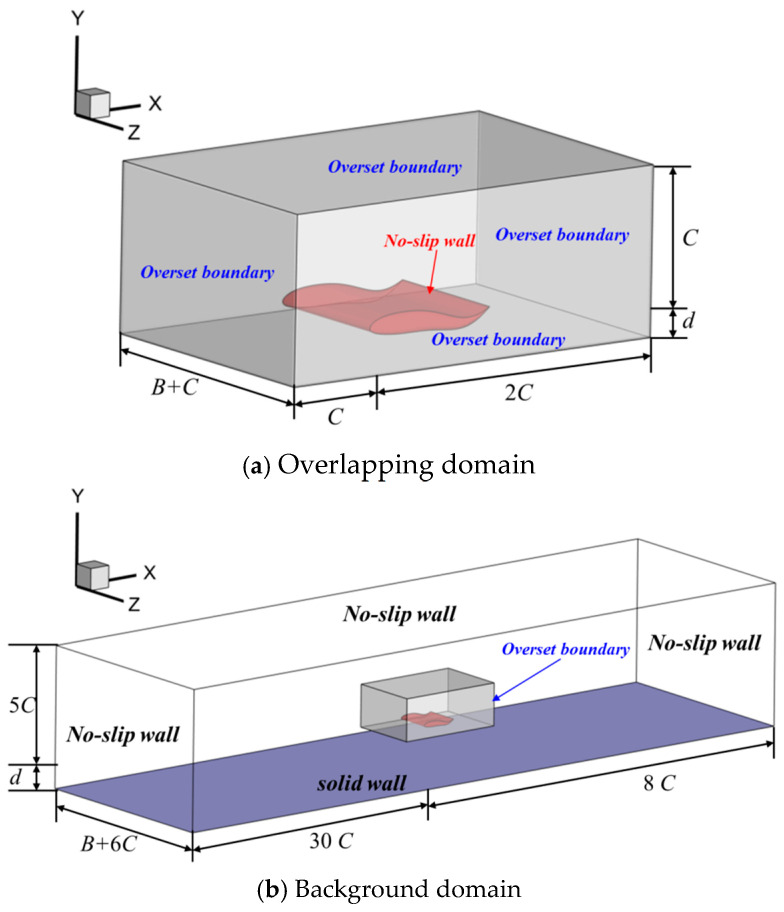
Diagram of computation domain for 3D model.

The computation domain in the current study consists of two parts: the background domain and the overlapping domain, respectively. As for the 2D condition (Seen in Figure 3), two rectangular-like domains were designed. Considering that the purpose of the current study was to investigate the ground wall effect, the length along the vertical direction should also vary with the wall distance. The geometric size of the overlapping domain was set as (x, y) = (3*C*, *d* + *C*), while the relevant size for the background domain was (*x*, *y*) = (38*C*, d + 5*C*). When it came to the 3D condition, as shown in Figure 4, two cuboid-like domains were designed, and the geometric size for the overlapping domain was designed as (*x*, *y, z*) = (3*C*, *d* + *C*, *B* + *C*). The corresponding size in the background domain of the 3D condition was (*x*, *y*, *z*) = (38*C*, *d* + 5*C*, *B* + 6*C*).

Concerning the boundary condition, the overset boundary condition was imposed on the surrounding edges (2D model) or faces (3D model) in the overlapping domain. The no-slip boundary condition was applied on the surface of the foil, and the mathematical formulation was expressed as $\frac{\partial p}{\partial n}=0,u=0$. As for the surrounding edges or faces of the background domain, the no-slip boundary condition was employed to simulate a water tank without the inlet and outlet. 

**Figure 5:**
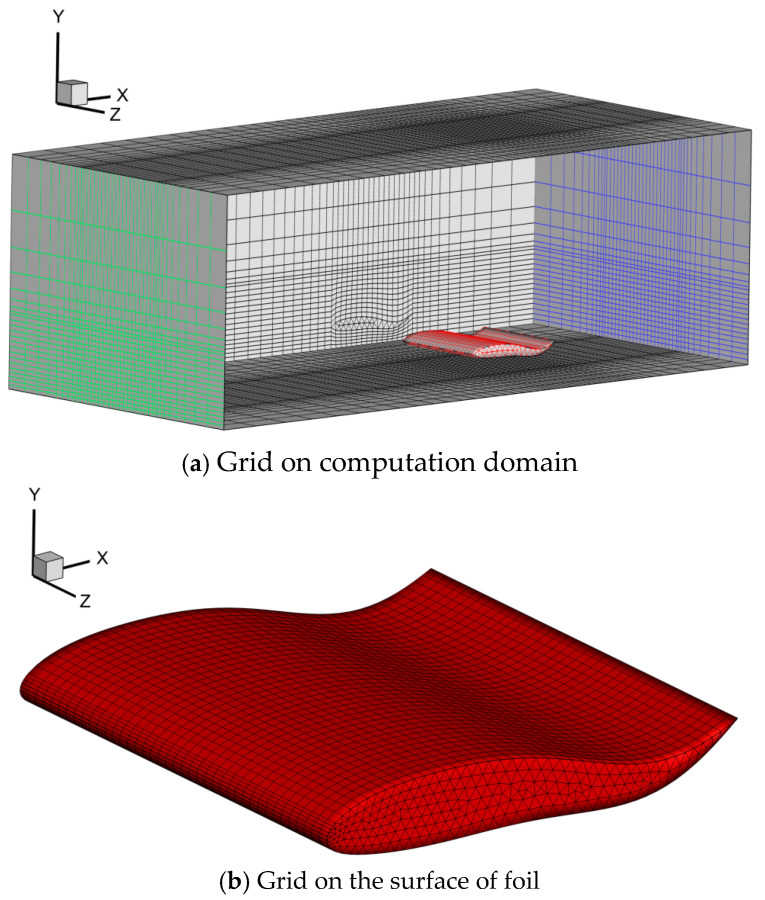
Computation grid for 3D model.

When it comes to the grid design, as seen in Figure 3, Figure 4 and Figure 5, the full-structured grid was employed to improve the calculation accuracy and efficiency. The local refine technique was also adopted to capture the flow structure.

### 3.3. Computation Procedure

**Figure 6:**
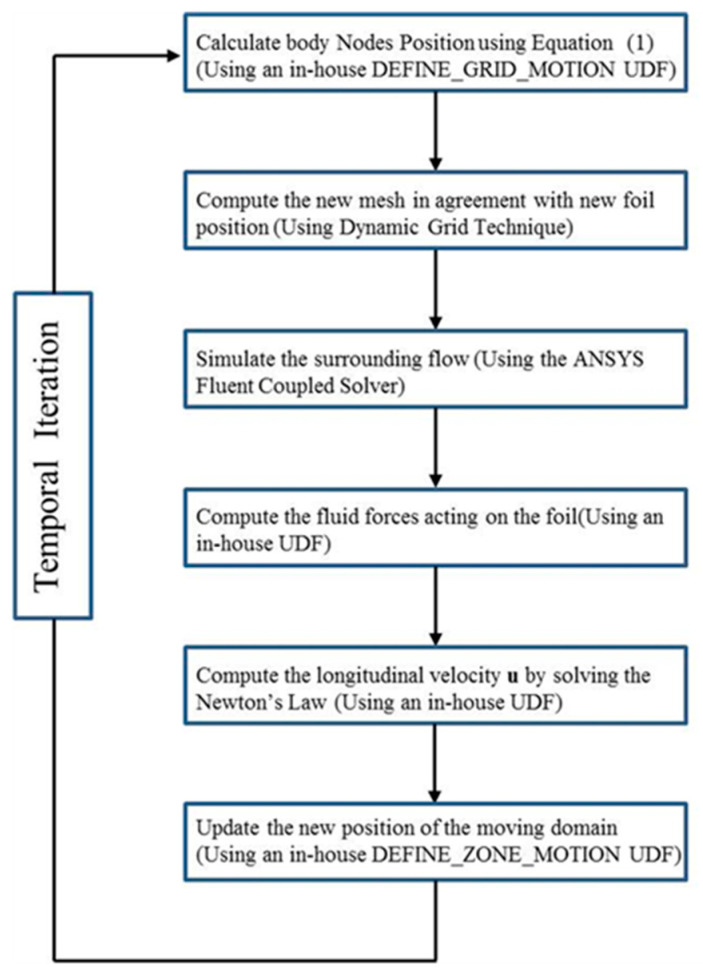
Diagram of solve procedure.

The current study involves the flexible foil’s positive deformation and the passive forward motion. In order to realize that several UDFs (User-Defined Functions) are written and embedded in the main solver of Fluent, the solve procedure is presented in Figure 6. As stated above, the flexible foil undergoes consistent travelling wavy motion all the time and the dynamic grid technique is employed to realize that. When the new position of foil is specified, the flow field around the foil is solved by the Fluent solver, and the relevant fluid dynamics acting on the foil are then acquired. In addition, Newton’s Second Law is adopted to calculate the corresponding value of acceleration at that time step. The calculation formula is expressed in Formula (4), where *F* is the fluid force, m is the mass of foil, and U denotes the moving velocity along the x direction. After that, the first-order explicit scheme is adopted to acquire the moving velocity of the foil at the current time step, as seen in Formula (5), where *F*_x_ is the horizontal force along the horizontal direction and *U*_t_ and *U*_t−Δt_ denote the moving velocity of the foil at the current and forward time step, respectively. Since the moving velocity is specified, the new position of the foil can be updated with the help of DEFINE_ZONE_MOTION. Until then, the calculation in the current time step is finished, and the calculation is repeated until the convergence criterion is satisfied. In the current study, if the difference in the foil’s fluid dynamics between two adjacent motion periods was less than 0.1%, the convergence could be considered satisfied.

(4) $$\sum F=m\frac{dU}{dt}$$

(5) $$U^{t}=\frac{F_{x}^{t-\Delta t}}{m}\Delta t+U^{t-\Delta t}$$

In addition, some hydrodynamic parameters are defined in Formula (6) to Formula (9), where *F*_x_ and *F*_y_ denote the drag force and lift force of the foil, and the corresponding mean value is denoted as *F*_x-mean_ and *F*_y-mean_. The mathematical formulation can be expressed as $F_{x}=\oint (-pn_{x}+\tau_{xi}n_{i})ds$, $F_{y}=\oint (-pn_{y}+\tau_{yi}n_{i})ds$, where *p* is the pressure, the indices *i* = 1, 2, 3 denote the *x*-direction, *y*-direction, and *z*-direction, respectively (repeated indices imply summation), *n*_i_ represents the *i*-th component of the unit normal vector on an element *dS*, and $\tau_{ij}$ is the viscous stress tensor. The propulsive efficiency of the foil is the ratio of the output power (*P*_out_) and input power (*P*_in_). The input power can be calculated as the product of velocity (*v*_n_) and pressure (*p*) integrated along the foil’s surface. The output power is defined as the product of *T*_mean_ and *U*_mean_, where *T*_mean_ and *U*_mean_ represent the mean value of the thrust force and forward velocity. It is noted that the relation between the thrust force and drag force is defined as *T* = −*F*_x_.

(6) $$F_{x-Mean}=\frac{1}{T}\int_{nT}^{(n+1)T}F_{x}dt,\;F_{y-Mean}=\frac{1}{T}\int_{nT}^{(n+1)T}F_{y}dt$$

(7) $$P_{in}=\oint_{\Gamma }v_{n}pdl$$

(8) $$\eta =P_{out}/P_{in}$$

(9) $$P_{out}=T_{Mean}\cdot U_{Mean}$$

### 3.4. Sensitivity Test

In this subsection, we describe the sensitivity test concerning the grid number and time step that was conducted. As for the 2D condition, five sets of grids and three time steps were selected. The number of grids was designed as *N* = 12,653, 25,400, 50,542, 101,520 and 204,500, respectively. The value of the time step was set as Δ*t* = *T*/1000, *T*/2000, and *T*/4000, respectively. The magnitude of U_mean_ and *η* under various values of the grid number and time steps are listed in Table 1. The relevant parameters were set as *a*_0_ = 0.02, *a*_1_ = −0.08, *a*_2_ = 0.16, *f* = 1, *λ* = 1.0. The turbulent model was set as the k-ω SST model. The specified boundary condition mentioned above remained unchanged when conducting the sensitivity test.

It can be seen in Table 1 that when the grid number is quite small (No. 1 to No. 3), the relative difference in the value of U_mean_ and *η* is large, and the maximum value of difference can reach 7.41%. With the further increase in the grid number (No. 4 and No. 5), the corresponding value of the difference is less than 0.2%, and we may conjecture that the further increase in the grid number will exert little influence on the value of U_mean_ and *η*. Therefore, we may conjecture that a further increase in the grid number can do little good in improving the calculation accuracy while exerting such a burden on computation cost. Therefore, the fourth set of grids was chosen, which could maintain a good balance between the computation cost and accuracy.

Similarly, when the time step alters from Δ*t* =*T*/1000 to Δ*t* =*T*/2000 (No. 4 and No. 6), the relative differences in U_mean_ and *η* are 3.02% and 4.27%, respectively. With the further decrease in time steps (No. 4 and No. 7), the relevant value of difference was less than 0.2%. Therefore, the time step is set as Δ*t* =*T*/2000 in the following calculation. Concerning the 3D condition, similar to the 2D condition, the sensitivity test results under five sets of grids and three kinds of time steps are listed in Table 2. The relevant motion parameters are identical to those of the 2D condition.

Similarly to that of Table 1, when the grid number reaches 8,762,520 (No. 4), the relative difference in the value of U_mean_ and *η* is too tiny to be observed, lying in the range of [0.12%, 0.18%]. Hence, the fourth set of grids was selected for the following calculation. For the same reason, the corresponding difference between Δ*t* = *T*/2000 and Δ*t* = *T*/4000 in the magnitude of U_mean_ and *η* was also smaller than 0.2%, meaning that the current time step (Δ*t* = *T*/2000) was suitable for the calculation.

### 3.5. Validation Test

In this subsection, the validation test of the current numerical method is carried out. Considering that both the 2D and 3D models are involved in the current study, there is a necessity to validate the corresponding accuracy separately. Here, three kinds of turbulent models were selected and they are the SST k–ω model, k–ε model, and S-A (Spalart–Allmaras) model, respectively.

**Figure 7:**
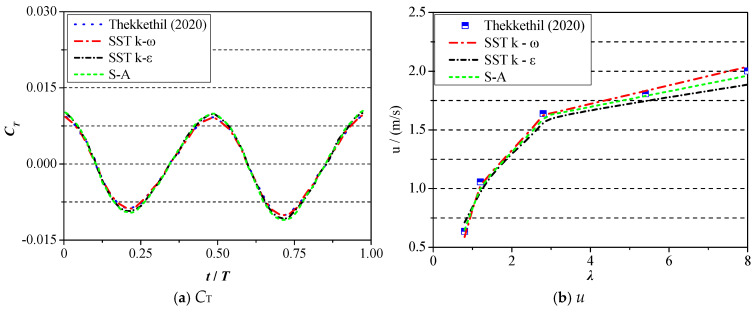
Validation of test result of 2D condition [24].

Concerning the 2D model, taking the results from Reference [24] as an example, the physical model in Reference [24] is a 2D NACA0014 foil, and the motion equation is identical to the current study. The validation test result is shown in Figure 7, where the variation curves of the thrust force coefficient are presented in Figure 7a, and the curve of forward velocity with respect to wavelength is exhibited in Figure 7b. The relevant motion parameters are set as *a*_0_ = *a*_2_ = 0, *a*_1_ = 0.1, *f* = 1, *λ* = 0.8. It can be vividly seen from Figure 7 that the overall changing tendency of the thrust force coefficient under three kinds of turbulent models agrees well with the results in Ref. [25]. However, when it comes to the value of moving velocity, the relative difference exhibits another phenomenon. When the wavelength lies in a small range [0.8–2.5], the relative difference is within 0.8%, and the results of the SST k–ω model present the best accuracy. With the further increase in wavelength, the relative difference between the results of the SST k–ω model and Ref. Reference [24] moves around 1.1%, while the corresponding value of relative difference under the other two turbulent models is pretty large (more than 12.5%). In view of that, the SST k–ω model will be adopted in the following calculation of the 2D condition.

**Figure 8:**
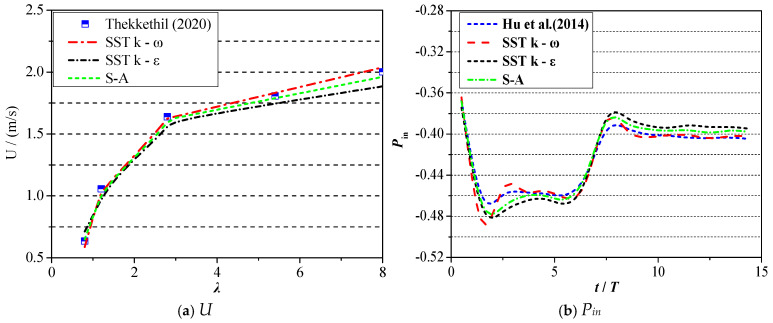
Validation of test result of 3D condition [24,25].

Similarly, the results from Ref. Reference [25] were employed to verify the accuracy under the 3D condition. The calculation model in Ref. Reference [25] is 3D foil with the oval section plane, and the aspect ratio is set as 4.0, and the ratio between the foil’s density and fluid’s density is 4.0. The comparison results are shown in Figure 8, where Figure 8a exhibits the variation curve of forward velocity with respect to wavelength. Figure 8b presents the comparison results of input power in the system. It can be seen in Figure 8, similar to the 2D condition, that the results of the SST k–ω model match well with those of Ref. Reference [25] and the corresponding difference lies in the range of [0.2%, 0.8%]. Therefore, the SST k–ω model is adopted in the following calculation of the 3D condition.

## 4. Results and Discussion

### 4.1. Effect of Ground Wall on Propulsive Property of 2D Foil

**Figure 9:**
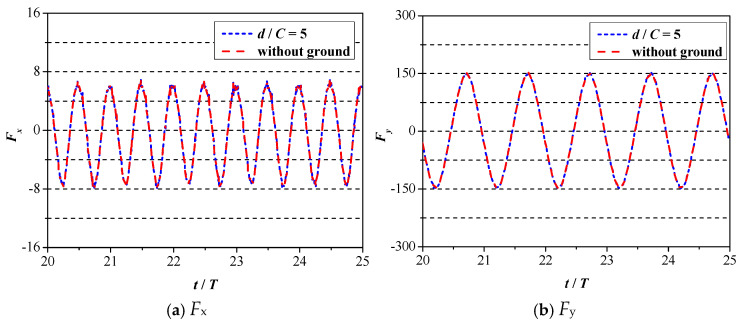
Variation curves of fluid dynamics under the condition of *d*/*C* = 5.0 and no ground wall.

In this subsection, the effect of the ground wall on the propulsive property of 2D foil is analyzed. Before starting the research, the critical distance, denoted as *d*_∞_, needs to be specified. The meaning of critical distance *d*_∞_ denotes the boundary distance. When the distance between the ground and foil is larger than that value (*d* > *d*_∞_), the effect of the ground wall can be ignored. In other words, the fluid dynamic of foil under the distance of d∞ is equal to that of an unbounded flow. As shown in Figure 9, the fluid dynamics of foil under the condition of *d*/*C* = 5.0 and no ground wall are plotted, whereas the variation curves of *F*_x_ and *F*_y_ are plotted in Figure 9a,b, respectively. It can be clearly seen from Figure 9 that two curves almost collapse with each other, meaning that when the ground wall distance is larger than *d*/*C* = 5.0, the effect of the ground wall can be ignored. In the following study, the emphasis will be placed on the condition of *d*/*C* < 5.0. 

**Figure 10:**
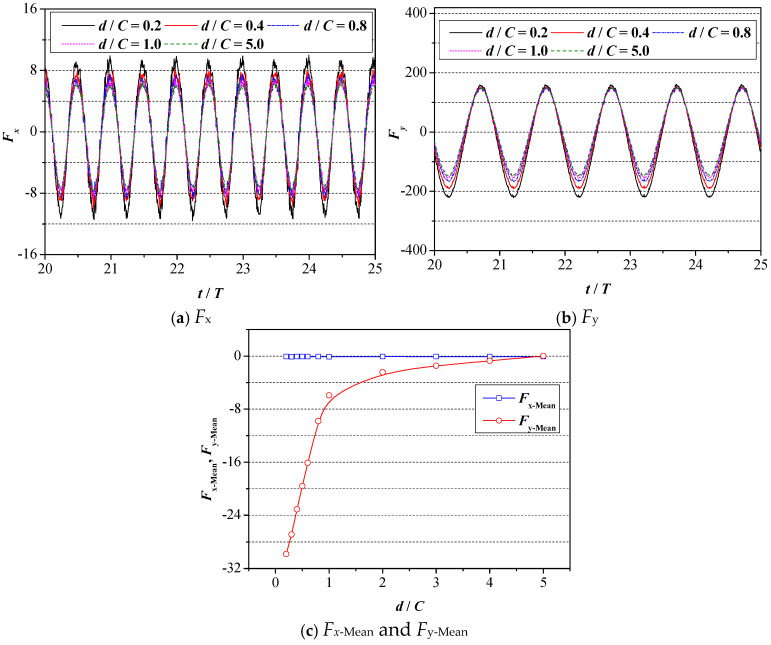
Variation curves of 2D foil’s fluid dynamic performance under various values of *d*.

The variation curves of 2D foil’s fluid dynamic performance under various values of *d* are plotted in Figure 10, where the drag force and lift force of 2D foil under various values of *d* are plotted in Figure 10a,b, respectively. The corresponding mean values of the drag force and lift force are summarized in Figure 10c.

Concerning the drag force, as seen in Figure 10a, the variation curve of the drag force at a certain ground distance exhibits periodic change, and there exist two peak values and valley values in one motion period, meaning that the changing period of the drag force is half of the motion period. With the continuous decrease in the ground wall distance, both the peak value and valley value exhibit the tendency to grow, and the smaller the wall distance is, the more distinct the growing trend is. In addition, it can also be obtained from Figure 10a that the growing magnitude of the peak value is almost equal to the magnitude of the valley value, which leads to the corresponding mean value being close to zero. The conclusion can also be verified in Figure 10c.

When it comes to the lift force, as seen in Figure 10b, the variation curve at a certain ground distance also exhibits periodic change, and only one peak value and valley value show up in a complete motion period, meaning that the changing period of the lift force is identical to that of motion period. Apart from that, it can be clearly seen from Figure 10b that the variation curves of the lift force at *d*/*C* = 5.0 are almost symmetrical with respect to the *x*-axis, namely the corresponding mean value should be equal to zero. With a further decrease in the ground distance, the corresponding symmetry was disrupted; namely, that the variation curve of the lift force is no longer symmetric to the *x*-axis. Specifically, the peak value of the lift force remains almost unchanged while the corresponding magnitude of the valley value keeps diminishing, and the smaller the value of the wall distance is, the greater the reduction. As for the corresponding value of C_L-Mean_, as seen in Figure 10c, the consistent decrease in the wall distance results in a continuous decrease in the lift force and the enlargement of asymmetry.

**Figure 11:**
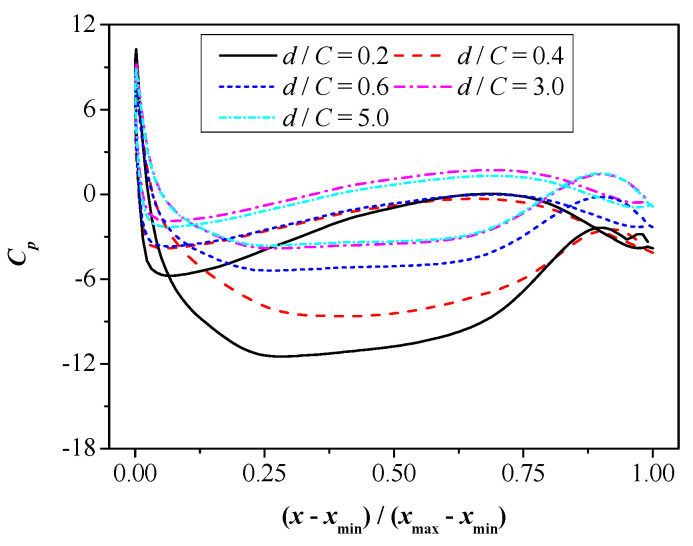
Distribution curves of foil surface pressure coefficient under various values of d.

In order to explain the numerical results in Figure 10, the distribution curves of the foil surface pressure coefficient under various values of *d* are plotted in Figure 11, and the motion time is set as *t*/*T* = 25.

As shown in Figure 11, the decrease in the wall distance leads to the consistent growth of the foil’s pressure difference, which may explain why the corresponding lift force increases consistently. This conclusion is consistent with that in Figure 10b. Specifically, when the distance alters from *d*/*C* = 5.0 to *d*/*C* = 3.0, although there exists an increase in the foil’s pressure, the increasing magnitude is relatively small. Therefore, the corresponding lift force coefficient increases slowly. The variation in the pressure distribution leads to a difference in the corresponding pressure difference between the upper and lower surfaces of the foil. The component of pressure difference along the vertical direction is the lift force, which explains why changes in pressure can affect the lift. With the further decrease in wall distance, in particular, when the distance alters from *d*/*C* = 0.4 to *d*/*C* = 0.2, the pressure on the upper surface of the foil remains almost unchanged while the relevant value on the lower surface keeps decreasing. Hence, the pressure difference between the upper surface and lower surface also presents a sharp increase, leading to the enlargement of the value of the lift force.

**Figure 12:**
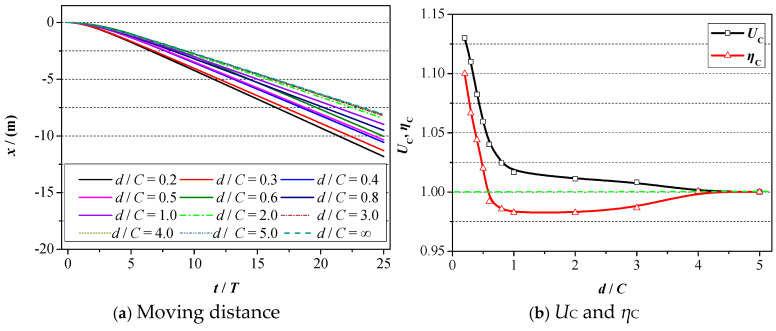
Propulsive property of 2D foil under various ground wall distances.

Next, the ground wall effect on the propulsive property of 2D foil will be analyzed, and the corresponding results are plotted in Figure 12. Specifically, the moving distance of foil with respect to motion time is plotted in Figure 12a, where *x* denotes the abscissa of foil’s gravity. The values of relative propulsive efficiency (*η*_C_) and moving velocity (*U*_C_) are summarized in Figure 12b. It is worth noting that in order to more obviously demonstrate the ground wall effect on the propulsive property, the value of propulsive efficiency and moving velocity under the condition of *d*/*C* = 5 is denoted as the reference value, and the corresponding values under other wall distances are equal to the ratio of the reference value.

It can be seen in Figure 12a that the moving distance of 2D foil presents a tendency to increase with respect to the motion time at certain wall distances. At the initial time of the foil’s motion, the curves of the moving distance increase very closely, and as the motion time goes on, the acceleration of the foil gradually increases, and the corresponding distance presents a tendency to increase sharply. With the further increase in motion time, the value of acceleration remains almost unchanged, and the relationship between the moving distance and motion time is almost linear. When the wall distance decreases, two distinct changes in the variation curve of the moving distance show up. For one, the decrease in the wall distance accelerates the speed of curve growth, namely the thrust force exerted on the foil, increases significantly, which can be confirmed in Figure 10. For another, the decrease in the wall distance enables the foil to move further. Hence, one conclusion can be drawn that the existence of a ground wall is beneficial for the foil to move more quickly and faster. 

Regarding the propulsive efficiency and moving velocity, as illustrated in Figure 12b, a decrease in the wall distance results in an increase in moving velocity. Specifically, when the wall distance alters from *d*/*C* = 5 to *d*/*C* = 2, the increasing magnitude and speed are quite small. When it comes to *d*/*C* < 1, the ground wall effect is much more distinct, and the corresponding moving velocity is also quite large. For example, when the value of the distance reduces from *d*/*C* = 1 to *d*/*C* = 0.2, the value of *U*_C_ alters from 1.02 to 1.13, and the increasing magnitude reaches 13%. Apart from that, it can also be acquired from Figure 12b that there almost exists a linear relationship between *U*_C_ and *d* in the range of d = [0.2, 1]. Concerning the propulsive efficiency, the value of *η* presents a tendency to first increase and then decrease with the consistent decrease in the wall distance. Specifically, when the distance lies in the range of *d*/*C* = [1, 5], the value of *η* keeps dropping when the wall distance decreases, meaning that the existence of the ground wall is not favourable for the propulsive efficiency during that range. With the further decrease in d (*d*/*C* < 1), the value of *η* starts increasing and when the value of *d*/*C* = 0.2, the value of η increases to 1.105. In summary, the existence of the wall can improve the forward speed and efficiency of foil, with a maximum increase of 13% in moving velocity and 10.5% in propulsive efficiency when the distance equals *d*/*C* = 0.2.

**Figure 13:**
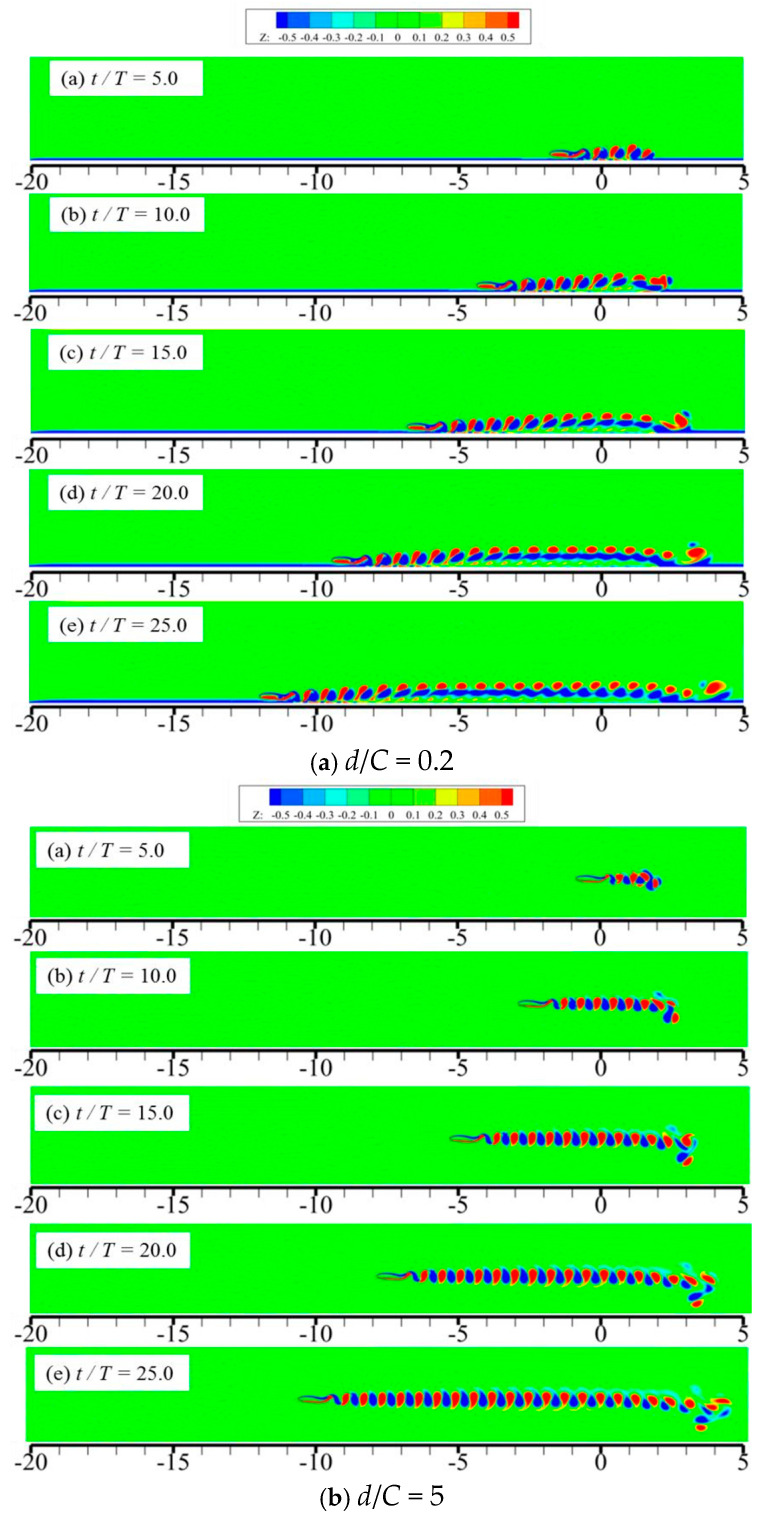
Vortex structures of 2D foil under various motion times.

Figure 13 presents the vortex structures of 2D foil under various motion times, where Z denotes the vortex magnitude along the *z*-axis direction and the corresponding definition is expressed as Z=∂u/∂y−∂v/∂x. As seen in Figure 13, at the initial time of the foil’s motion, two kinds of vortex structures with different rotation directions emerge on the upper and lower surfaces of the foil. With the further motion of the foil, the detached vortex structures start falling off, and the vortex ring structure is formed. As is known from Figure 12, the acceleration and moving velocity of the foil at the initial time are pretty low; therefore, the distance between the two adjacent vortex rings is also very small. With the further increase in motion time, the vortex structures in the wake of the foil are stretched, and the distance between two adjacent structures is also enlarged. Apart from that, there also exist many differences in vortex structures when the wall distance keeps decreasing. Specifically, when *d*/*C* = 0.2 (as seen in Figure 13a), the vortex structures shedding from the foil start to emerge with the vortex structures on the surface of the ground wall and move together along the flow direction, forming a flat structure.

**Figure 14:**
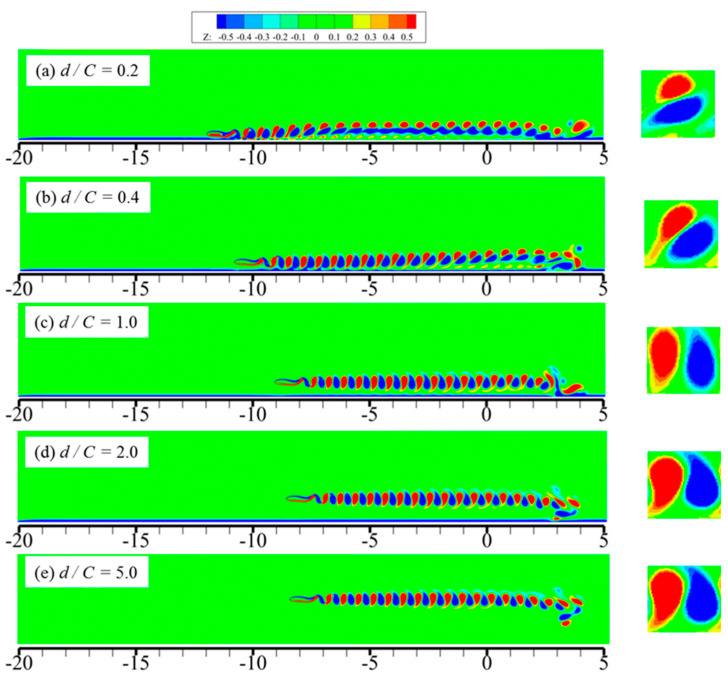
Vortex structures of 2D foil under various ground wall distances.

In order to further explain the results obtained above, the vortex structures of 2D foil under various ground wall distances are shown in Figure 14, and the motion time is selected as *t*/*T* = 25. The enlarged view of the vortex structures is plotted in the right part of the figure. 

As seen in Figure 14, when *d*/*C* = 5.0, the effect of the ground wall can be ignored, and the flow field can be approximately considered as unbounded flow. The vortex ring in the wake of the field is equipped with a regular shape, and the corresponding centerline is almost parallel to the flow direction. With the ground wall distance starting to decrease, the vortex ring presents a tendency of right-upper inclination, and the smaller the distance is, the more the corresponding incline angle will be. Since the rotation direction of the two adjacent vortex structures is opposite, a backward jet will be formed, coupled with the released energy. The released energy can be decomposed into horizontal and vertical directions, and the energy along the horizontal direction is favourable for the foil’s forward motion. It can be clearly seen in the enlarged view of Figure 14 that when the distance alters from *d*/*C*= 5 to *d*/*C*= 1.0, the corresponding vortex ring starts to tilt towards the vertical direction. The relevant released energy along the horizontal direction is reduced, leading to a drop in propulsive efficiency. However, when the distance alters from *d*/*C* = 1.0 to *d*/*C* = 0.2, the vortex ring presents a trend of horizontal inclination. More energy is released along the horizontal direction, which may explain why the propulsive efficiency increases correspondingly. 

### 4.2. Effect of Ground Wall on Propulsive Property of 3D Foil

In this subsection, the ground wall effect on the propulsive property of 3D oscillating foil will be analyzed. The aspect ratio of 3D foil is set as *AR* = 1.0, and the corresponding motion parameters are identical to those of 2D foil.

**Figure 15:**
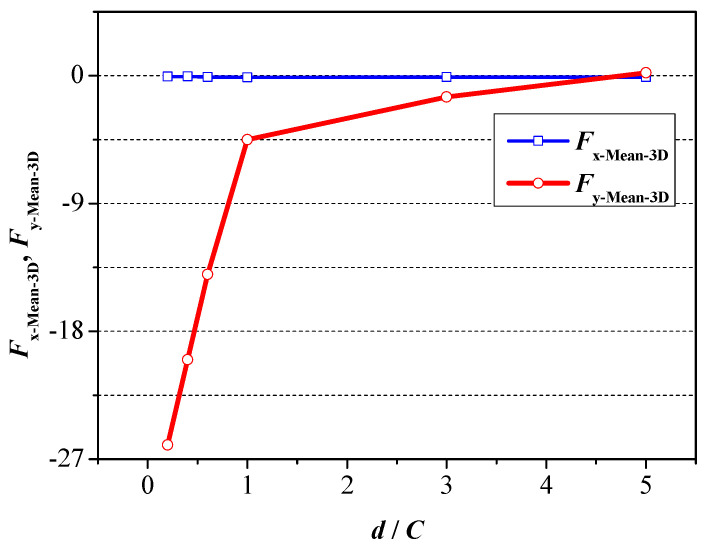
Mean value of drag force and lift force under various ground wall distances.

The mean value of drag force and lift force under various ground wall distances is plotted in Figure 15. It can be seen from Figure 15 that, similar to 2D foil, the mean value of the drag force is close to zero, meaning that a mostly stable moving velocity will be acquired when adopting the travelling wave motion. Moreover, the consistent decrease in the wall distance results in a gradual increase in the mean value of the lift force, which means that the existence of a ground wall destroys the balance along the vertical direction.

**Figure 16:**
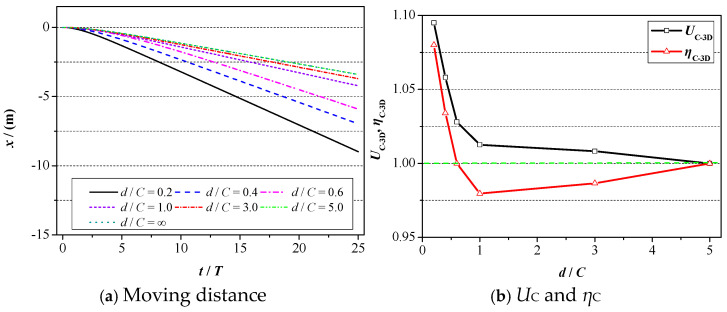
Propulsive property of 3D foil under various ground wall distances.

The propulsive properties of 3D foil under various ground wall distances are given in Figure 16. It can be seen in Figure 16a that the moving distance increases very slowly at the initial time, and when the moving velocity of the foil reaches a stable state, the moving distance increases linearly with respect to motion time. In addition, the decrease in wall distance improves the moving velocity and little motion time is required to obtain a stable state. This phenomenon is similar to that of the 2D condition.

**Figure 17:**
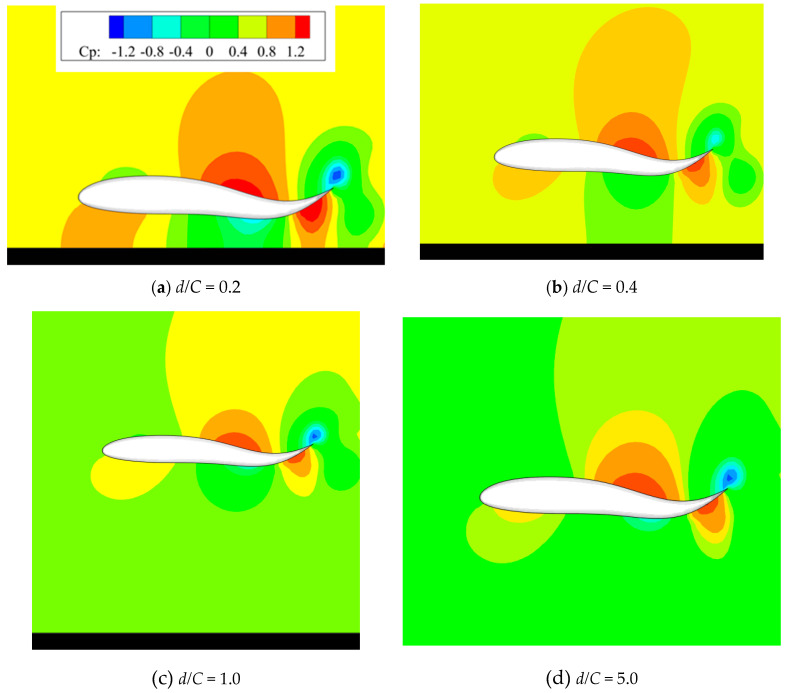
Cloud map of pressure distribution in the longitudinal section of 3D foil under various wall distances.

When it comes to moving velocity, as seen in Figure 16b, the effect of the ground wall on the propulsive property of 3D foil can be decomposed into two parts. When the value of the distance falls within the range of *d*/*C* = [1, 5], there is a slight increase in the moving velocity, approximately 1.0%. With the further decrease in the wall distance, when *d*/*C* < 1, the increasing speed climbs to 9.6%, which is a little lower than that of 2D foil (12.8%), meaning that the sensitivity to the ground effect of 3D foil is lower than that of 2D foil. A cloud map of pressure distribution in the longitudinal section of 3D foil under various wall distances is presented in Figure 17. It can be seen from Figure 17 that the decrease in the wall distance enlarges the pressure difference of 3D foil. The component of pressure difference along the horizontal direction is favourable for the foil’s forward motion. That may explain why the decreased wall distance is beneficial to the foil’s forward motion.

Concerning the propulsive efficiency, as seen in Figure 16b, the effect of the ground wall can also be divided into two phases. When the wall distance lies in the range of *d*/*C* = [1, 5], the decrease in the wall distance leads to a small reduction in the value of *η* and the highest reduction amplitude can reach 2.1%. With a further decrease in the wall distance (*d*/*C* < 1), the trend turns to the opposite side; the magnitude of *η* climbs quickly with the decrease in the wall distance; and the peak increasing value can reach 10.5%. In summary, the existence of the ground wall can improve the forward speed and efficiency of 3D foil, with a maximum increase of 9.6% in moving velocity and an increase of 2.1% in propulsive efficiency when the distance equals *d*/*C* = 0.2.

**Figure 18:**
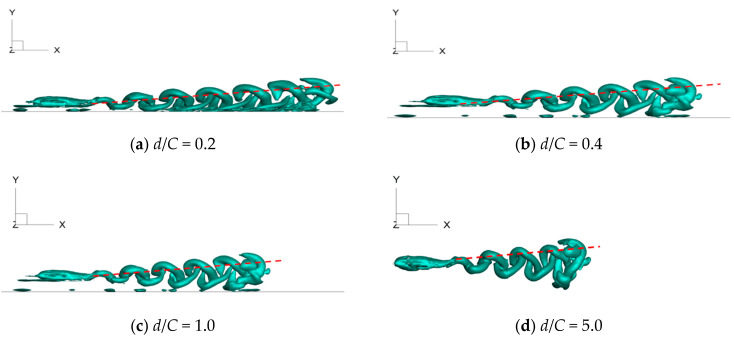
Side view of vortex structures under various values of wall distance.

**Figure 19:**
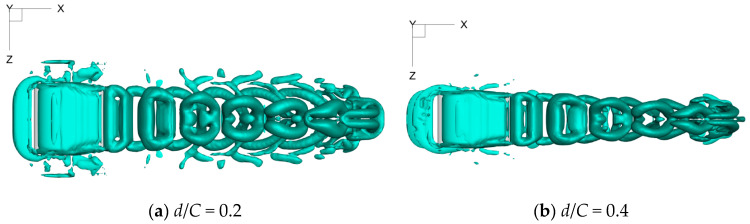

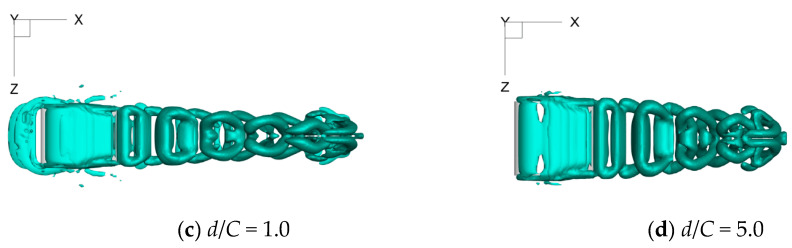
Top view of vortex structures under various values of wall distance.

**Figure 20:**
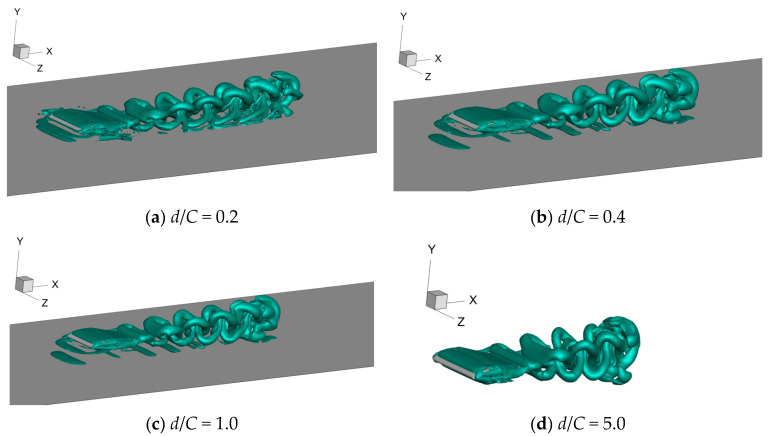
Perspective view of vortex structures under various values of wall distance.

The typical vortex structures of 3D foil under various values of wall distances are captured and presented in Figure 18, Figure 19 and Figure 20. Specifically, the side view, top view, and perspective view are shown in Figure 18, Figure 19 and Figure 20, respectively. The corresponding motion time is selected as *t*/*T* = 6.0; the λ_2_ vortex criterion [26] is employed to characterize the vortex structures; and the corresponding value is fixed as λ_2_ = −1.0.

As seen in Figure 18, at certain wall distances, the vortex structures on the upper and lower surfaces of 3D foil fall off from the foil under the impact of the foil’s self-propulsion, and a staggered vortex street is formed. There exists an angle between the centerline of the vortex street and the horizontal direction, called an oblique angle (*θ*). As stated above, the released energy is often accompanied during the formation of the vortex ring, and the energy can be decomposed into the horizontal and vertical direction. The horizontal direction is beneficial for the propulsion of the foil. Hence, the oblique angle can serve as an index for evaluating the propulsive property of the foil. As shown in Figure 18, the corresponding values of the oblique angle are θ = 17.4°, θ = 18.0°, θ = 16.1°, and θ = 11.5°, respectively, when the wall distance alters from *d*/*C* = 5.0 to *d*/*C* = 0.2. The magnitude of the oblique angle exhibits a tendency to decrease sharply when the foil moves closely towards the ground wall. The small value of the oblique angle corresponds to the large amounts of energy along the horizontal direction, which may explain why the existence of a ground wall is beneficial for the foil’s self-propulsion.

Moreover, it can also be seen from Figure 19 and Figure 20 that the span length of the vortex structure keeps reducing during the process of the foil’s forward motion, which is consistent with that of the constrained mode state [27]. When the distance alters from *d*/*C* = 5.0 to *d*/*C* = 1.0, the corresponding span length reduces quickly, and much energy is dissipated, leading to a decrease in propulsive efficiency. However, as the wall distance decreases further, the vortex structure’s span length exhibits a gradual growth tendency. In particular, when *d*/*C* = 0.2, the span length of the vortex structure is larger than the foil’s span length, and the energy dissipation reaches its lowest value, which is why the value of *η* climbs to the peak.

## 5. Conclusions

The effects of the ground wall on the propulsive property of auto-propelled foil have been analyzed, and the research object focused on 2D and 3D foil. The overlapping grid and dynamic mesh technique were employed to realize the foil’s positive deformation and passive forward motion. The main conclusions drawn in the current study are given as follows.

1. The effect of the ground wall on the fluid dynamics of oscillating foil is tremendous. Specifically, the existence of the ground wall will break down the symmetry of the lift force, and the positive value of the lift force will increase correspondingly. As for the drag force, both the peak and valley values will increase due to the wall effect.
2. The ground wall will also significantly influence the foil’s propulsive properties. As for the moving velocity, the existence of a ground wall is beneficial for the foil’s forward motion, and the closer the ground wall is, the faster the foil will move. Concerning the propulsive efficiency, the value of *η* descends to the valley value and then climbs with a consistent decrease in the wall distance.
3. There exists a close connection between the foil’s property and the surrounding flow structures. As for the 2D foil, the existence of the ground wall results in the small distance between the two adjacent vortex structures as well as the small oblique angle of the vortex ring. In the case of 3D foil, the ground wall not only reduces the oblique angle but also increases the span length of the vortex structure, thereby reducing energy consumption.

The current UUV has some disadvantages, such as high noise and low propulsive efficiency, which limit the further application of UUV. In view of this, inspired by the good propulsive properties of natural creatures in nature, such as whales or dolphins propelled by travelling wavy motion, a novel conceptual design of bionic UUV has been designed. The bionic UUV can glide near the seabed to monitor the environment and scan the terrain. Compared with gliding in an unbounded flow, the fluid dynamics of travelling wavy foil undergo a huge difference when gliding near the ground wall. Therefore, there is a great necessity to investigate the ground effect on the propulsive properties of travelling wavy foil. The conclusions acquired can provide theoretical guidance for the controlling and navigation of bionic UUV. 

## Figures and Tables

**Table 1:** Sensitivity test results for 2D condition.

*No.*	*N*	Δ*t*	U_Mean/_(m/s)	*η* (%)
1	12,653	T/2000	0.682	67.8
2	25,400	T/2000	0.635	62.8
3	50,542	T/2000	0.628	61.2
4	101,520	T/2000	0.610	60.5
5	204,500	T/2000	0.609	60.4
6	101,520	T/1000	0.629	63.2
7	101,520	T/4000	0.609	60.4

**Table 2:** Sensitivity test results for 3D condition.

*No.*	*N*	Δ*t*	U_mean/_(m/s)	*η* (%)
1	608,120	T/2000	0.582	60.2
2	1,696,270	T/2000	0.552	56.5
3	4,784,860	T/2000	0.538	52.4
4	8,762,520	T/2000	0.533	52.2
5	13,468,500	T/2000	0.532	52.1
6	8,762,520	T/1000	0.545	53.3
7	8,762,520	T/4000	0.534	52.31

## Data Availability

All relevant data are within the paper.
